# Effect of Qing’e formula on the in vitro differentiation of bone marrow-derived mesenchymal stem cells from proximal femurs of postmenopausal osteoporotic mice

**DOI:** 10.1186/s12906-015-0777-2

**Published:** 2015-07-24

**Authors:** Bo Shuai, Lin Shen, Rui Zhu, Piqi Zhou

**Affiliations:** Department of Integrated Traditional Chinese and Western Medicine, Union Hospital, Tongji Medical College, Huazhong University of Science and Technology, Wuhan, China

**Keywords:** Osteoporosis, Bone marrow-derived mesenchymal stem cells, Qing’e formula, Transforming growth factor β

## Abstract

**Background:**

Qing’e formula (QEF), prepared from an ancient Chinese recipe, was previously suggested to regulate bone metabolism and improve bone mineral density in patients with osteoporosis. To study the effects of medicated serum containing QEF on the in vitro differentiation of bone marrow-derived mesenchymal stem cells (BMSCs) isolated from the proximal femurs of postmenopausal osteoporosis (PMOP) mice.

**Methods:**

Using an established mouse model of PMOP, mononuclear cells were isolated from the bone marrow present in the proximal femurs and cultured. PMOP mice were also randomly divided into four groups: the untreated group (Group A) and the groups treated with respectively low (Group B), medium (Group C), and high (Group D) concentrations of QEF. Serum was isolated from each and used to treat the cultured BMSCs in conjunction with recombinant human bone morphogenetic protein-2 (rhBMP-2). Cell morphology, proliferation rates, intracellular alkaline phosphatase (ALP) activity, and transforming growth factor-beta 1 (TGF-β1) mRNA expression were evaluated.

**Results:**

QEF-treated serum, particularly that containing moderate and high concentrations, appears to enhance the rhBMP-2-mediated changes in cell morphology, proliferation, and differentiation (determined via the expression of TGF-β1 mRNA and ALP activity) observed in the BMSCs isolated from PMOP mice.

**Conclusions:**

QEF may play a role in the prevention and treatment of PMOP by enhancing the activity of rhBMP-2.

## Background

Postmenopausal osteoporosis (PMOP) is one of the major diseases seriously affecting female health worldwide. PMOP is categorized as a kind of bone atrophy disease affecting, as the name suggests, postmenopausal women. The current understanding of how to treat this disease is largely limited. Notably, some success in the prevention and treatment of PMOP has been observed using a traditional Chinese medicine (TCM) known as Qing’e formula (QEF). QEF consists of four primary compounds: cortex eucommiae, fructus psoraleae, semen juglandis, and Allium sativum. These ingredients are blended in a specific ratio and the resulting mixture has been used in TCM since the Song Dynasty (10th century CE) to invigorate the kidneys, replenish bone and muscle tissue, slim the body, and improve complexion. QEF is also commonly used clinically as an oestrogen agonist for the treatment of osteoporosis, particularly in PMOP [1].

Furthermore, our previous research has shown that by adjusting the mRNA expression of vitamin D3 receptor (VDR), QEF is capable of activating bone metabolism to prohibit further loss of bone mass, thereby preventing the bone deterioration observed to occur during osteoporosis [2]. Notably, VDR has been shown to regulate a number of downstream target genes important in mineral metabolism as well as many essential genes involved in osteoblast function [3]. For example, transforming growth factor-beta 1 (TGF-β1) is locally produced by osteoblasts and plays an important role as a “coupling factor” in bone remodelling. The link between VDR and TGF-β1 was discovered in a study by Takeshita et al. [4] in which they demonstrate a synergistic effect between 1-alpha, 25-dehydroxy-vitamin D3 (i.e., a VDR-dependent mechanism) and TGF-β1 on the transcriptional activation of activation protein-1 (AP-1), a known regulatory factor in bone metabolism. Although it is possible that QEF functions via a similar signalling cascade, the underlying mechanism between QEF and VDR, or any of these other osteoporosis-related genes, during PMOP is largely unknown.

With the rapid development of various molecular biology techniques, our understanding of bone atrophy-related diseases, including PMOP, has greatly expanded. In fact, recent studies suggest that the differentiation and regulation of bone marrow-derived mesenchymal stem cells (BMSCs) play an important role in osteoporosis prevention and treatment. Thus, in this study, we have utilized serum isolated from QEF-treated PMOP mice to explore the effects of QEF on the osteogenic differentiation of BMSCs isolated from the proximal femurs of PMOP mice. In doing so, we sought to elucidate the fundamental cellular and molecular mechanisms of PMOP and uncover the possible clinical applications of QEF.

## Methods

### Main instruments and reagents

The following instruments were used in this study: flow cytometer (Becton-Dickinson, USA), double-sided ultraclean workbench (China), carbon dioxide incubator (Sheldon, USA), inverted phase contrast microscope (Olympus, Japan), and a UV spectrophotometer, (Tianjin TianGuang Optical Instruments Co., LTD.). The primary reagents used in the following experimental procedures were: LG-DMEM powdered medium (Gibco, USA), fetal bovine serum (Gibco, USA), trypsin powder (Sigma, USA), alkaline phosphatase (ALP) assay kit (Shanghai Hongqiao Medical Reagent Research Institute), β-glycerol sodium phosphate (Sigma, USA), and recombinant human bone morphogenetic protein 2 (rhBMP-2) (R&D, USA).

### Preparation of QEF

Four kinds of herbal QEF were purchased from the Pharmacy of Union Hospital at Tongji Medical College of Huazhong University of Science and Technology (HUST). The main ingredients of QEF, which include cortex eucommiae (960 g), fructus psoraleae (480 g), semen juglandis (300 g), and Allium sativum (240 g), were washed and placed in a multifunctional extractor (Table 1). All four herbs were identified and authenticated by Prof. Jiancai Wu (The Botany and Drug Department of Union Hospital at Tongji Medical College of HUST). The herbs were immersed in five-fold their volume of regular water for 2 h, followed by boiling for 2 h. After filtering the boiled decoction, the herbal residues were repeatedly boiled twice for 1 h each time, and the resulting boiled decoction was filtered, combined with the first filtrate, and condensed to a thick paste. This paste was then added to a three-fold volume of 95 % ethanol while stirring. After standing for 24 h, the solution was filtered to recover the ethanol fraction, which was then concentrated to a decoction of 5 g/mL [5].

**Table 1:** Components of modified QEF and total dose of each herb

No. herbal name	Processing	Contents (g)
1 cortex eucommiae	steep in wine	960
2 fructus psoraleae	fried, peeled and dipped in ginger	480
3 semen juglandis	peeled	300
4 Allium sativum	steaming	240

### Preparation of serum containing QEF

Female C57BL/6 mice were purchased from and maintained at the Experimental Animal Center of HUST, Wuhan, China (certificate: NO. 0237269; Hubei provincial experimental animal facility permit: SYXK [Hubei] 2010-0057). Disinfected food and water were provided under sterile conditions.

A mouse model of PMOP was established for experimental use by Thompson et al. [6]. Briefly, the animals used in this study were anaesthetised with an intraperitoneal injection of 1 % pentobarbital sodium (30 mg/kg body weight (b.w.)) and maintained in a prone position. Ovariectomy was performed via a midline dorsal incision under sterile conditions after which both ovaries were identified adjacent to the inferior pole of the kidneys in the peritoneal cavity, the blood vessels were tied off using No. 4 sutures, and both ovaries were removed. The layers of tissue were then closed and sutured. Starting from post-operative day 5, a pap smear (once/day) was taken from each animal for 5 consecutive days. A successful bilateral ovariectomy was confirmed by the absence of keratosis in these pap smear screenings. Samples exhibiting keratosis were discarded. Animals were fed and allowed to move ad libitum for 12 weeks [6].

The ovariectomised mice were randomized into four groups (*n* = 10 for each): group A (blank control group treated with 2 ml of distilled water); group B (low concentration group treated with 0.85 g/kg b.w. QEF), group C (medium concentration group treated with 1.7 g/kg b.w. QEF), and group D (high concentration group treated with 3.4 g/kg b.w. QEF). Further, the mice in each group received a daily intragastric dose of the designated treatment (water or various concentrations of QEF) via gavage for 3 days. The medium dose of QEF was calculated in accordance with the guidelines correlating dose equivalents between humans and laboratory animals, which is based on the ratio of the animal’s body surface area [7]. Blood samples were collected 2 h after the final administration.

Serum was then isolated from each blood sample, and heat inactivation was conducted at 56 °C for 30 min. After filtration and repackaging, these “medicated serum” samples were stored at −80 °C for future use. Notably, all experiments were performed in accordance with HUST’s ethical guidelines for animal care as well as the guidelines set by the World Health Organization, and the experimental protocols were all approved by the animal care committee of HUST.

### Isolation, cultivation, and identification of BMSCs

Bone marrow (4–5 ml) was isolated from untreated PMOP mice and placed into a 20 ml sterile centrifuge tube containing Dulbecco’s modified Eagle’s medium (DMEM). Percoll (1.073 g/ml) was used to separate the mononuclear cell fraction using density gradient centrifugation. After several rounds of washing, the mononuclear cells were re-suspended in DMEM containing 10 % fetal bovine serum, and incubated at 37 °C with 5 % CO_2_. The medium was changed every 3–5 days, and non-adherent cells were removed in order to obtain cells with the known characteristics of adherent BMSCs. Cell morphology, adherence, and cellular growth were observed every day. After cell confluence was 80–90 %, 0.25 % trypsin (containing 0.02 % EDTA) was used to passage the cells. The trypsinized cells were then inoculated into new 25 mL culture flasks at a density of 5,000 per cm^2^ and incubated. The culture medium was changed in these flasks once every 2 to 3 days. These cells were marked as P1 (the first generation). The passaging procedure outlined above was repeated each time the cells reached 80–90 % confluency. Further, when the P3 cells achieved 90 % confluency, the culture media was removed from each flask and the cells were thoroughly washed twice using phosphate buffered saline (PBS). After washing, aliquots of the cells (1 × 10^6^ cells) were incubated in fluorescence-activated cell sorting (FACS) buffer containing monoclonal antibodies against CD34, CD90, CD44, CD45, or an appropriate isotype control antibody (all obtained from Boshide, China). After 30 min in the dark on ice, cells were washed again in FACS buffer. Then, the expression of CD34, CD90, CD44, and CD45 antigens were detected via flow cytometric analysis using CellQuest software. Cells were gated on forward and side scatter to exclude debris and aggregates, and dead cells were excluded using 7-Amino-Actinomycin D (Boshide, China) staining.

### BMSC treatment and morphological observation

Serum isolated from mice in the four experimental groups was added to separate culture wells, each of which also contained recombinant human bone morphogenetic protein-2 (rhBMP-2, 150 ng/mL). Third generation BMSCs were collected and the cell density was adjusted to 1 × 10^5^/ml. For each group, 20 μl of the cell suspension was added and incubated at 37 °C with 5 % CO_2_. According to results from preliminary experiments, no cytotoxicity was detected when the concentration of animal serum in the culture medium was below 20 % [8]. Therefore, in this study, animal serum isolated from each group was diluted to a concentration of 10 % in DMEM prior to further use. Morphological changes in the BMSCs for each group were observed using an inverted phase contrast microscope.

### Determination of relative cell number and cell proliferation levels

Cell proliferation was determined for the BMSCs in each of the four treatment groups using the MTT method on days 1, 3, 5, 7, 10, 12, 15, and 20. To each well, 20 μl of MTT solution (5 mg/mL) was added and incubated for 4 h at 37 °C before the reaction was terminated. The supernatants were then carefully aspirated off each well. DMSO (150 μl) was added and cells were lysed after 10 s of oscillation, which fully dissolved the crystals. Optical density (OD) values were determined with a spectrophotometer at a wavelength of 490 nm.

### Detection of ALP activity

On days 5, 10, 15, and 20, the cultured cells of each treatment group were washed twice with PBS, and 0.25 % trypsin was used to digest the cells. This trypsinisation was followed by the addition of 1 ml of culture medium to terminate the digestion. After centrifugal washing, cell lysis solution was added, and ALP activity was assayed according to the kit manufacturer’s instructions. OD was measured with a spectrophotometer at a wavelength of 520 nm and ALP activity was calculated.

### Determination of TGF-β1 mRNA expression

Total RNA was extracted from three unique cultures of each of the four treatment groups using TRIzol protocol. The isolated mRNA was reverse transcribed using the following reaction mixture: 9 μL of H_2_O, 1.0 μL of oligo (dT15), 2.0 μL of template, 4.0 μL of 5× reaction buffer, 2.0 μL of 10 mM dNTPs, 1.0 μL of RNA inhibitor, and 1.0 μL of reverse transcriptase. The following reaction conditions were used: 42 °C for 30 min and 80 °C for 5 min. Using the subsequent cDNA from each sample, TGF-β1 mRNA expression was then assessed using quantitative RT-PCR with FastStart Universal SYBR Green 1 PCR master mix (Rox, Roche, USA) in an ABI7300 real-time PCR system (Invitrogen, China). The following forward (F) and reverse (R) primers were used: β-actin (F: TCACCCACACTGTGCCCATCTACGA, R: TCACCCACACTGTGCCCATCTACGA); and TGF-β1 (F: GTGCTCGCTTTGTACAACAGC, R: TTACCAAGGTAACGCCAGG). The amount of TGF-β1 mRNA was normalized to that of β-actin mRNA. The quantitative PCR reaction mixture contained 1.0 μL of cDNA, 5.0 μL of 10× reaction buffer, 7.0 μL of 25 mmol/L MgCl_2_, 1.0 μL of 10 mmol/L dNTPs, 0.8 μL of forward primers (20 pmol/μL), 0.8 μL of reverse primers (20 pmol/μL), 1.0 μL of SYBR Green, and 0.5 μL of Taq polymerase (5 U/μL). The cycling conditions were as follows: 95 °C for 10 min, followed by 40 cycles of 95–60 °C for 15 s, and 60 °C for 5 min. Finally, the Ct values were calculated using the ΔΔCt method and analysed as previously described [9].

### Statistical analysis

All data are presented as the mean ± standard deviation (SD). Statistical analyses were performed using the Kruskal-Wallis test or an analysis of variance (ANOVA) followed by the least significance difference test (Student’s *t*-test) for multiple comparisons using SPSS software (version 13.0, SPSS, Inc., Chicago, IL, USA). A *P* value less than 0.05 was considered statistically significant.

## Results

### Identification of BMSCs

**Fig. 1:**
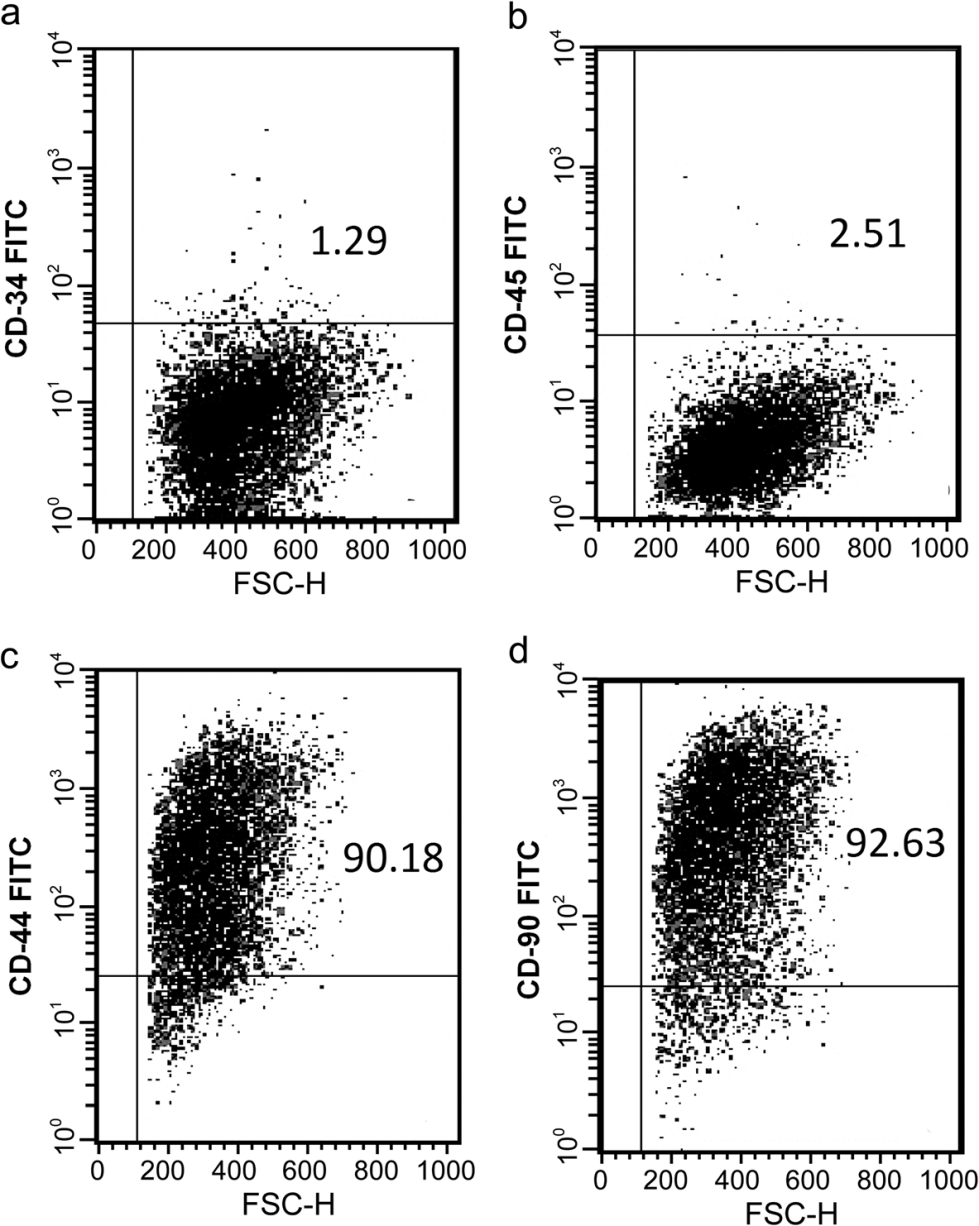
Identification of BMSCs. Flow cytometric detection data showing little to no expression of the negative cell markers CD34 (**a**) and CD45 (**b**) as well as the positive expression of the BMSCs markers CD44 (**c**) and CD90 (**d**). *FITC* fluorescein isothiocyanate, *FSC*-*H* forward scatter-hight

Using flow cytometry, the main negative BMSC surface markers CD34 and CD45 were confirmed to be negative in our cell cultures, being expressed in only 1.29 and 2.51 % of the cells, respectively (Fig. 1a and b). On the other hand, the cultured BMSCs were observed to express the main positive markers CD44 and CD90 on the cell surface, being present on the surfaces of 90.18 and 92.63 % of the cells, respectively (Fig. 1c and d). Notably, when culturing cells isolated directly from a tissue, it can often be difficult to prevent the contamination of other cell types, particularly after continued passaging. These higher percentages (more than 90 %), indicate that our BMSC cultures primarily contained this specific cell type and contamination from other mononuclear cells was low.

### BMSC growth and morphology following the addition of QEF-treated serum

**Fig. 2:**
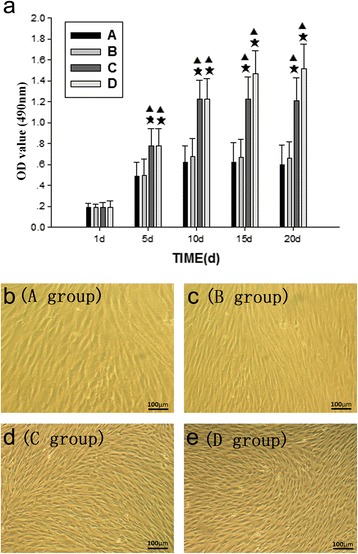
Proliferation and morphology of cultured BMSCs after treatment with QEF-containing serum. The level of cellular proliferation, determined by counting the number of cells, was visually monitored in cells cultured in serum treated with water (control, group *A*); a low concentration of QEF (group *B*); a medium concentration of QEF (group *C*); and a high concentration of QEF (group *D*). During this time, there was no significant difference observed between groups *A* and *B* (*P* > 0.05), while groups *C* and *D* were significantly higher than *A* and *B* from day 5 onward (*P* < 0.05) (**a**). Values are expressed as the mean ± SD; ^★^ *P* < 0.05 compared to group *A* at the specified time point, ^▲^ *P* < 0.05 compared to group *B* at the specified time point. All groups reached a growth plateau between culture day 10 and 11. Representative images of the cellular morphology at day 10 are shown for group *A* (**b**), group *B* (**c**), group *C* (**d**), and group *D* (**e**). All photomicrograph images were obtained at a ×100 magnification

Although the number of cells increased in all four treatment groups, the level of cellular proliferation in groups A and B, as determined by the change in the number of adhesive cells, was distinctly slower than that observed for groups C and D (Fig. 2a). Further, there was no significant difference between groups A and B in terms of the relative number of cells (*P* > 0.05), while the number of cells in groups C and D were significantly higher than those of groups A and B from day 5 onward (*P* < 0.05). Cellular growth in groups A, B, and C peaked on day 10 before entering the plateau phase, while that of group D reached a peak value on day 11.

Moreover, the morphology of the cells in groups A and B also appeared to be different compared to that of groups C and D. For example, after composite cultivation for 4 h, cell adhesion was observed for groups C and D, and the cells appeared to be oval or round in morphology, while less cells adhesion was apparent in groups A and B at this time. Furthermore, after one day in culture, the proliferating cells in groups C and D were uniformly distributed and spindle-shaped. On day 3, as the number of cells increased in each group, the cells in groups C and D appeared to be polygonal or spindle-shaped, unlike the cells of groups A and B. As the cells continued to proliferate from day 5 to 10, the cells in groups C and D clearly occupied the flask with a monolayer of morphologically similar cells (Fig. 2b–e).

### Effect of QEF-treated serum on ALP activity and TGF-β1 mRNA expression in cultured BMSCs

**Fig. 3:**
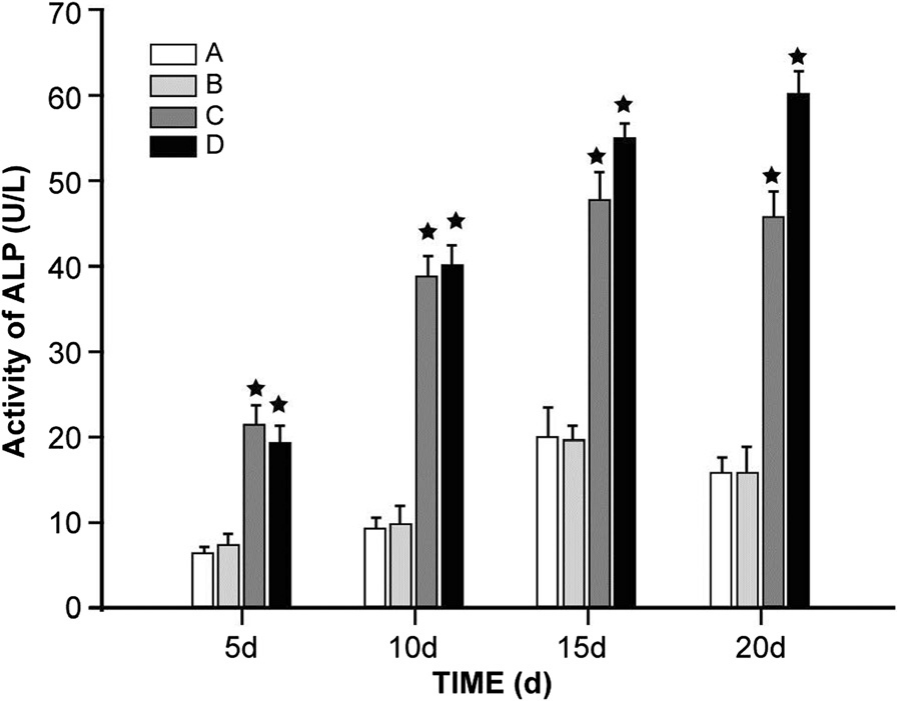
ALP activity assay. Cellular ALP activity was monitored in cells cultured in serum treated with water (control, group *A*); a low concentration of QEF (group *B*); a medium concentration of QEF (group *C*); and a high concentration of QEF (group *D*). Activity appears to gradually increase in each group for the first 15 days in culture, with the ALP in groups *C* and *D* being significantly (*P* < 0.05) more active than that of groups *A*. This difference is even more evident from day 15 to day 20, as the ALP activity in groups *A* and *B* decreased slightly while that of *C* and *D* remained high. The values are expressed as means ± SD; ^★^ *P* < 0.05 compared to group *A* at the specified time point

Over time, the cellular ALP activity in the cultured BMSCs gradually increased in each treatment group until day 15 (Fig. 3). Notably, the cellular ALP activity in group B was not significantly different from that of the control (group A) at any time point, while the activities observed for groups C and D were significantly higher (*P* < 0.05). This was particularly evident after day 15, when the cellular ALP activity of groups A and B appeared to gradually slow down, while those of groups C and D continued to increase.

**Fig. 4:**
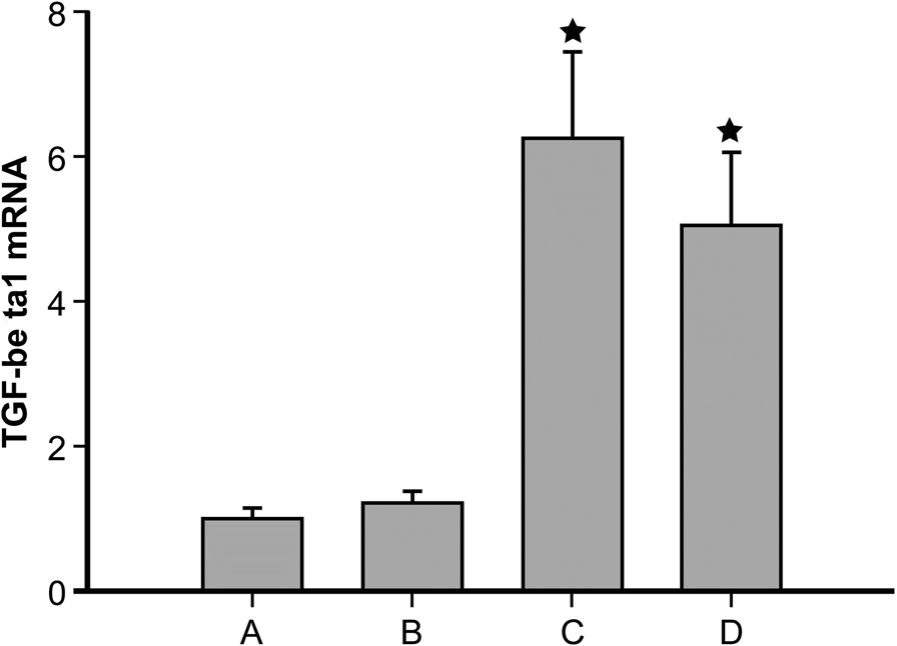
TGF-β1 mRNA expression. The relative expression of TGF-β1 mRNA was evaluated in cultured BMSCs 20 days after the introduction of serum treated with water (control, group *A*); a low concentration of QEF (group *B*); a medium concentration of QEF (group *C*); and a high concentration of QEF (group *D*). The expression observed in group *B* (1.21 ± 0.18) was not significantly different from group *A* (1.00 ± 0.16, *P* > 0.05), while the expression in groups *C* and *D* (6.23 ± 1.21 and 5.03 ± 1.01, respectively) were significantly higher than those of group *A* (*P* < 0.05). Values are expressed as the mean fold change ± SD, with the control group being set to 1; ^★^ *P* < 0.05 compared to group *A*

In addition to monitoring ALP activity, the relative expression of TGF-β1, another marker of osteoblast mesenchymal cell differentiation, was also evaluated 20 days after QEF-treated serum was introduced to the cultured BMSCs (Fig. 4). The relative TGF-β1 mRNA expression levels observed for groups C (6.23 ± 1.21) and D (5.03 ± 1.01) were significantly higher than those of the control group A (1.00 ± 0.16, *P* < 0.05), while the expression in group B (1.21 ± 0.18) was not significantly different from group A (*P* > 0.05).

## Discussion

In the present study, we have utilized primary in vitro culture of BMSCs isolated from mice with induced PMOP in order to investigate the possible clinical application of QEF for the treatment of this disease. It is has been well documented that after menopause, bone resorption occurs at a faster rate than bone deposition/growth, causing a gradual weakening of the bone. This can result in an increased occurrence of bone fracturing and a significantly altered quality of life for the patient. Although the specific mechanism underlying PMOP is unknown, a number of treatment options have been explored. For example, in osteoporosis and osteonecrosis patients, QEF treatment was shown to elevate the levels of serum matrix metalloproteinase-2, bone ALP, osteocalcin, cross-linked C-telopeptide of type I collagen, and urinary cross-linked N-telopeptide of type I collagen [2, 10]. These data indicate that QEF could potentially enhance osteoblastic activity, promote bone formation, inhibit bone resorption, and increase bone mass and quality. However, the mechanism underlying these changes or even the specific cell type(s) affected in the bone was previously unexplored. To our knowledge, this is the first published report describing the effects of QEF on the growth and morphology of BMSCs isolated from PMOP-induced mice.

BMSCs are known to differentiate into various types of cells, including osteoblasts, fibroblasts, chondroblasts, and adipocytes [11]. Further, spontaneous in vitro differentiation of BMSCs into stable osteoprogenitor cells is largely dependent on the culture conditions [11–15]. Therefore, optimal in vitro conditions are of great importance when studying the formation of bone tissues [16–18]. These primary BMSC cultures have also been used to investigate the cellular changes that occur in response to experimental treatment of a bone disease, including osteoporosis [19]. Notably, in almost all experiments utilizing primary BMSC cultures, including the current study, cellular differentiation was induced using one or more BMPs. BMPs are glycoprotein polypeptides that are innately expressed in the bone matrix. The members of this protein family all have similar structures and functions (with the exception of BMP-1), contain a disulfide bond, and have a molecular weight between 18 and 30 kDa [20]. Although they function in a similar manner to induce osteogenic mesenchymal cell differentiation into cartilage and bone, the ability of each BMP to do so greatly varies. It appears that BMP-2 has the strongest osteogenic capability and is a crucial regulator of bone tissue formation in the human body [21]. Therefore, this BMP has been widely studied and used during in vitro BMSC culture experiments [22].

Notably, in preliminary experiments, QEF-containing serum did not have any significant effects on the function of BMSCs isolated from PMOP mice when used without rhBMP-2 (data not shown). Thus, we chose to focus on the synergistic effects mediated by QEF in rhBMP-2-induced BMSCs isolated from the proximal femurs of PMOP mice. Using these BMP-induced cell cultures, we have shown that in the first 10 days following treatment with QEF-treated serum, the cells treated with moderate (group C) and high (group D) concentrations of QEF grew exponentially, while cells from the untreated controls (group A) and low concentration groups (group B) displayed slower growth. Further, from day 10 to day 20, the growth became completely stagnant in the control, low concentration, and moderate concentration groups, while the cells in the high concentration group still appeared to grow, although at a lower rate compared to earlier time points. Further, cells cultured in QEF-treated serum were also more adhesive and displayed morphological characteristics of healthy differentiated osteoblastic cells, indicating that the moderate and high concentrations of QEF exhibit a significant effect on BMSCs condition during PMOP.

In order to further determine the effects of QEF on BMSCs of PMOP mice, an ALP activity assay was conducted. ALP, a zymoprotein secreted by osteoblasts, is highly specific and its expression level in osteoblasts can reasonably reflect the degree of differentiation and functional status of the cell [23]. The results of this analysis show that, within 15 days, ALP activity in the groups treated with moderate and high concentrations of QEF was rapidly increased, while that observed for the low concentration and control groups was only marginally increased. Further, after this first 15 days in culture, the ALP activity in the low concentration and moderate concentration groups as well as the control group stopped increasing altogether, while that of the high QEF concentration group still appeared to increase slowly. These results demonstrate that the cellular ALP secretion lagged behind cellular growth and differentiation and that a higher concentration of ALP was secreted after the mature cell was formed.

Furthermore, the expression of TGF-β1 mRNA was used as an additional marker of cellular differentiation. TGF-β1 is widely expressed in normal tissues and is particularly abundant in bone [24]. Indeed, a large number of studies have shown that TGF-β plays a pivotal role in bone regeneration and is involved in the formation and growth of bone during embryogenesis [25]. During the early stages of bone formation in newborns, TGF-β1 and TGF-β2 in particular are expressed in the cartilage and periosteum, and TGF-β1 has been shown to promote the proliferation and differentiation of mesenchymal cells into mature bone cells in these tissues [26]. Mechanistically, TGF-β1 mainly functions to regulate the transcription and activity of genes essential for mesenchymal cell differentiation in addition to promoting the synthesis of extracellular matrix (ECM) and ECM associated proteins (e.g., collagen, osteonectin, and osteopontin) and increasing the rate of bone matrix deposition [27]. TGF-β1 expression also influences the directional migration of osteoblasts, which is essential for the chemotactic movement of these cells towards the site of active bone resorption [28]. Notably, other in vitro studies have shown that a kidney-invigorating (Bushen) decoction used in TCM can enhance the expression of TGF-β1 in the cytoplasm and plasma membrane of osteoblasts [29, 30]. Here, our data demonstrate that rhBMP-2 alone (control group) can induce TGF-β1 expression, indicating BMSC differentiation. We also observed that this TGF-β1 expression was further promoted in QEF-treated cells, with the maximum effect being found for the cells cultured in serum treated with moderate and high concentrations of QEF.

Finally, it is important to note that there are several limitations to this study. First, the preparation and application of QEF-containing serum in this study are still at the exploration stage as there are no unified standards to follow in the current literature. Effective compounds used in TCM are largely underused in the majority of modern studies, making their optimization during in vitro analyses more difficult and time consuming. Second, the effects of rhBMP-2 and QEF on BMSCs isolated from normal, non-PMOP mice were not studied in the current investigation. However, we believe the side-effects of QEF on non-PMOP cells would be limited, particularly at the serum concentration utilized. Third, only a limited number of relevant indicators are included in this study, including ALP activity and TGF-β1 mRNA expression. Additional work using other indicators is necessary to further explore the cellular mechanisms involved in PMOP and the effects of QEF treatment.

## Conclusions

Taken together, this study is the first effort to determine the possible clinical application of QEF to treat PMOP. These experiments highlight the use of rhBMP-2 to induce the passage and differentiation of BMSCs isolated from the proximal femurs of PMOP mice into osteoblast-like cells. In addition, this process appears to be further promoted by culturing the cells in medicated serum isolated from PMOP mice treated with varying concentrations of QEF in a dose-dependent manner. While this study provides a secure foundation, additional work is warranted in order to fully understand the role of this TCM in osteoporotic BMSC differentiation.
